# The effectiveness of psychoeducation and problem-solving on depression and treatment adherence in adolescents living with HIV in Botswana: an exploratory clinical trial

**DOI:** 10.1186/s13034-022-00541-3

**Published:** 2023-01-04

**Authors:** Anthony A. Olashore, Saeeda Paruk, Adegboyega Ogunwale, Mkpang Ita, Andrew Tomita, Bonginkosi Chiliza

**Affiliations:** 1 Department of Psychiatry, Nelson R Mandela School of Medicine, University of KwaZulu-Natal, Durban, South Africa; 2 Department of Psychiatry, Faculty of Medicine, University of Botswana, Gaborone, Botswana; 3 Department of Clinical Services, Neuropsychiatric Hospital Hospital, Aro, Abeokuta, Nigeria; 4 Department of Forensic and Neurodevelopmental Sciences, Institute of Psychiatry, Psychology and Neuroscience, King’s College, London, UK; 5 Department of Psychology, Neuropsychiatric Hospital, Aro Abeokuta, Nigeria; 6 KwaZulu-Natal Research Innovation and Sequencing Platform, Nelson R Mandela School of Medicine, College of Health Sciences, University of KwaZulu-Natal, Durban, South Africa; 7 Centre for Rural Health, College of Health Sciences, University of KwaZulu-Natal, Durban, South Africa

**Keywords:** Psychoeducation, Problem-solving, Life-steps, Depression, Adherence, HIV-infected adolescents, Clinical trial

## Abstract

**Background:**

This study aimed to explore the effectiveness of psychological interventions (PI): psychoeducation, problem-solving, and rehearsal strategies on depression and adherence in HIV-infected adolescents in Botswana.

**Methods:**

Fifty adolescents living with HIV were randomized into control (n = 25) and intervention groups (n = 25), the latter being exposed to five weeks of PI sessions. The PHQ-9 and visual analog scale (VAS) were used to measure the outcomes: depression and adherence at pre-intervention, 5- and 24 weeks post-intervention.

**Results:**

The participants’ mean age (SD) was 17.38 years (1.1), the two groups being similar in socio-demographic variables: gender (χ^2^ = 2.22; p = 0.135) and age (U = 285, z =  − 0.55, p = 0.579). The intervention group scored significantly lower on depressive symptoms (PHQ-9 [F (1,50) = 12.0, p = 0.001, ƞ_p_^2^ = 0.20]) and higher on adherence score (VAS [F (1,50) = 13.5, p = 0.001, ƞ_p_^2^ = 0.22]) than the control group after 5 weeks. The post-hoc analysis showed that the significant improvements in depressive symptoms (z =  − 4.03, p < 0.01, r [effect size] = 0.88) and adherence (z =  − 4.05, p < 0.01, r = 0.88) at post-test in the intervention group were maintained at 24 weeks. This project was registered with ClinicalTrials.gov (NCT05482217).

**Conclusion:**

The 5-week PI showed promising effectiveness in addressing depression and adherence in adolescents living with HIV in Botswana.

## Background

Treatment adherence in adolescents remains a concern, with more than 60% reporting inconsistent adherence in a study conducted in South Africa [1]. Depression has been related to poor medication adherence in adolescents living with HIV (ALWHIV) [2] and is associated with various symptoms, such as poor judgment and concentration, as well as pseudodementia, which leads to poor treatment attitudes and health-seeking behaviors [2–4].

Poor adherence, or the suboptimal use of antiretroviral treatment (ART) has been linked to a high rate of drug resistance, the development of more virulent strains, an increase in the rate of progression to AIDS, and an upsurge of new HIV infection [5]. However, there are few studies on improving medication adherence among ALWHIV, most of which were conducted in developed countries [6]. The literature has described the benefit of psychological intervention (PI), such as cognitive-behavioral interventions: psychoeducation, problem-solving and rehearsal strategies, in a limited number of adult patients [7] and youth studies outside Africa [8]. Studies in Africa are therefore lacking, and it is unclear if these methods can be applied to adolescents in its different settings [9].

Various types of single-component adherence interventions with good results have been published. For example, one study among patients aged 4–24 years found improved adherence to pill swallowing technique training six months post-intervention [10]. Another study explored the benefit of text message reminders on adherence and found that this method significantly improved self-reported adherence among HIV-positive youth [11]. Shegog and colleagues [12] recruited 10 perinatally infected youth aged 14–22 years and exposed them to a theory-based program designed for youth with HIV. They were assessed for adherence before and after the exposure; the program consisted of tailored activities addressing attitudes, knowledge, skills, and self-efficacy related to ART adherence. A significant improvement was observed in knowledge about HIV, treatment uptake, and attitude towards taking the correct dose of their medication close to the appropriate time every day. These studies, however, did not consider the possible impact of psychosocial factors on adherence or use a comparison group. While most of these reports are exploratory and innovative, their applicability in African settings such as Botswana, which account for up to 70% of the  continents reported new HIV infection, remains unknown [9].

Some multicomponent psychological strategies for improving adherence in adults have also been published, such as that by Lyon and colleagues [13], who conducted a study among 23 youths aged 15–22 and their families. Participants received 12 biweekly family and youth education sessions and six biweekly youth-only sessions. In addition, at the end of the study, they introduced several adherence-enhancing devices, such as pillboxes, calendars, and watch alarms, at the youth-only counseling sessions. Of the 23 youth who participated in the study, 22 (91%) self-reported increased medication adherence after completion [13]. In a similar study, Safren and colleagues [7] described a cognitive-behavioral intervention comprising psychoeducation, problem-solving, and rehearsal strategies to address depression and adherence among adults with HIV infection. This 11-session “Life-steps” program included practicing adaptive pill-taking plans, reducing depressive symptoms, and increasing patients’ skills for adhering to HIV treatment.

Recently, researchers [8] adapted the Life-step strategies for adolescents and young adults, the ‘Positive STEPS’ strategy making use of five sessions instead of 11 and including other adherence-enhancing plans, such as short phone messages, alarms, and calendars. They also reported an improved level of adherence, as in previous studies [7, 13]. While Safren and colleagues reported on adults [7], although they included depression and adherence, Mimiaga et al. focused mainly on adherence without depression in adolescents and young adults [8]. At the time of writing, no study was found that had examined the effect of short psychological interventions on adherence in depressed ALWHIV to the best of the authors' knowledge.

Depression is highly comorbid with HIV, plays a major role in poor attitudes to medication use or prescribed therapy [14], and has been reported to interfere with retention in care, immunity, and the course of the illness [14–16]. Perhaps the noteworthy but less than optimal effects of existing adherence-promoting interventions are due to the interfering effects of psychosocial problems, such as depression, hence the need to explore those targeting the two related issues. It was hypothesized that PI effectively reduces depressive symptoms and ultimately improves adherence in ALWHIV. Therefore, this study aimed to explore the effectiveness of PI on depression and treatment adherence in HIV-infected adolescents in Botswana.

## Materials and methods

### Study design

This was a randomized, controlled trial to assess the psychological intervention program in ALWHIV, aged 15–19, who attend medical clinics at Botswana Baylor Children's Clinical Centre of Excellence (BBCCCE). An earlier manuscript has reported details regarding the study site and clinic activities [17].

### Study participants and eligibility criteria

The participants were adolescents living with HIV, aged 15 to 19, on ART for at least six months, able to communicate and follow instructions in English or Setswana, not psychotic, scored above ten on CAT rapid cognitive screen, and willing to participate. Alhough the WHO definition of adolescents (10–19 years) [18] was adopted in this study, those below the age of 15 were excluded based on ethical advice, as the presence of an adult caregiver is required [1]. Moreover, a study conducted in a similar setting suggested that older adolescents, such as 15 years and above, are more likely to be poorly adherent than children, younger adolescents, and adults [1]. In addition, participants should have met the criteria for depression, as indicated by the Mini International Neuropsychiatric Interview for Children and Adolescents (MINI-KID), these being: mild to moderate depression, with a score of 9–14, according to the PHQ-9; not be on psychotropic drugs, and be poorly adherent to ART, as assessed by the Visual Analogue Scale, with a score below 95 [19].

### Sampling method and selection

This study is the second phase of a survey to investigate the psychosocial barriers to good treatment adherence in two groups of ALWHIV in Botswana: the behaviourally and congenitally infected. The survey identified depression as the most significant barrier to good adherence, this being the basis for this second phase. In the first phase of the study, a minimum of 490 ALWHIV was targeted using the formula for comparing proportions in two equally sized groups [20], with 245 in each group. However, 622 participants were recruited from the clinic using a non-probability sampling method; recruitment taking place when suitable candidates came to the clinic and if they met the study criteria. This method was adopted because of the rare nature of the sample, especially those not born with the infection, and the reduced turnout of clinic patients due to COVID-19. In the study's second phase, a minimum sample size of 20 per group of intervention and control was determined using Kadam & Bhalerao formula for controlled clinical trials [21]. This sample was increased to 25 per group in anticipation of drop-outs. A two-stage random sampling method was adopted to select 50 participants from the sub-sample of the phase 1 survey study who met the inclusion criteria.

Of the 622 adolescents surveyed in the first phase of the study, 123 had mild to moderate depressive symptoms, were eligible for phase 2, and were informed of the possibility of being contacted for the second phase. They were each assigned an identification number that was linked to their contact details to conceal their identities, which the principal investigator kept and only used for selection and follow-up purposes, the contacts being deleted on completion of the study. The identification numbers of the 123 participants were entered into SPSS software to identify 50 who were contacted randomly. This process of generating numbers was repeated until 50 participants had been identified who were willing to participate, and once their primary caregivers had consented, they were enrolled. Of the 50, 10 could not be reached during this process, and three withdrew their consent, these 13 being replaced from the remaining pool of 73.

### Randomization and masking

In the second stage, the participants were contacted by phone and informed about the possibility of being allocated to either an intervention or control group. SPSS was used to randomly allocate them into two groups of 25 participants, and they were informed about how to participate using the same medium. Of the research team, which included psychologists, statisticians, and psychiatrists, only the principal investigator and the research assistant were aware of the randomization. The intervention’s outcome and detail were not discussed with either group so as not to introduce a bias, and they were requested not to discuss their allocation and treatment with friends. Also, to avoid therapeutic coercion, none of the staff attending to them was involved in the exercise, and the participants were duly informed of their right to disengage from the program without any consequences for their treatments.

### Data collection procedure

Written informed consent was obtained from those who were 18 and 19 years old, and parental consent and adolescent assent were obtained from those below the age of consent (less than 18 years) before commencing the study [22]. Consent was obtained at the beginning of phase I and repeated at the start of phase II. Ethical procedures and COVID-19 protocol were strictly followed, as recommended by the BBCCCE management.

Due to COVID-19, the intervention group participants were only attended to in a maximum group of five, with the 25, therefore, being divided into five groups labeled ‘A to F.’ While the first three groups completed the five sessions as five per group, one participant dropped out from the fourth group and two from the fifth group after the first session without prior notice. This necessitated the constitution of the sixth group with three new participants to make up the 25 participants in the intervention group; however, two groups met per week for five weeks. While the intervention group met weekly for five weeks, the controls were only given appointments for the fifth week after the baseline assessments, as per the standard of care.

To enable a comparison between the two groups, attention was paid to grouping participants by age, resulting in two age groupings: 15–16 years and 17–19 years, with two groups being the younger age group and three the older. This was done in accord with the recommendation of the ethical review board to enable identification and attention to age-specific needs, particularly in relation to sexual issues, since 16 is the legal age of sexual consent in Botswana [23].

The weekly meetings with the intervention groups were conducted at the BBCCCE using the same program for the two age groups to ensure consistency. The manualized PI program on treatment adherence used for the project was prepared by the team psychologist (MI) and the principal investigator (AO) and consisted of five structured sessions that were offered weekly, each lasting 60 mins. The five sessions were adapted from studies conducted elsewhere [7, 8], were delivered by a trained graduate psychology counselor and supervised by the principal investigator. The sessions involved identifying problems related to depression and adherence, such as stigma and peer and family-related issues, and guided participants on providing age-specific adaptive solutions.

### Adverse effects

While no adverse events were recorded, one participant, who was lost to follow-up from the intervention group, complained of suicidal ideation and reported at the clinic seven weeks after the end of the project.

### Intervention

The intervention involved interactive discussion, role-play, and brief plenary sessions in the local language, Setswana, mixed with English, which took ten minutes of the sessions. This intervention was adapted from intervention programs designed in high-income countries [7, 8, 13] to being locally relevant and adolescent-friendly by the study psychologist and the attending healthcare providers at the centers. Five minutes were spent on relaxation and exercise at the start of each meeting, followed by a review of the subject learned in the previous week to allow participants to share how they practiced their skills. There was a recapitulation of the discussion at the end of every session, with homework being given to enable them to practice the new skills. Finally, there was a recap of all the sessions at the end of the week five meeting, with participants being asked to rate each session on its usefulness and their level of satisfaction. The sessions were: introduction to program and rapport building, identifying and dealing with good adherence I and II, handling privacy, social support and handling slips and wrap up review.

#### Session 1: Introduction to program and Rapport building

This session entailed building rapport, setting ground rules to enhance confidentiality; and telling participants what the intervention sessions were about and what to expect during each session. Their knowledge of HIV, depression, stigma, and legal rights of ALWHIV were assessed, with basic knowledge about HIV, medication adherence, and its importance, being provided using South African video clips. Participants discussed these areas after viewing the videos and were introduced to the five steps of the problem-solving technique. The five steps consisted of  identifying the root of their problems, such as stressful or negative life events; generating potential solutions; choosing one solution; applying the chosen solution, and evaluating the results. The videos contained animation on how the virus enters the body and takes over the host system, and the medications help them fight the infection. The session concluded with an assignment for participants to apply the techniques to address depression and adherence.

#### Session 2: Identifying and dealing with barriers to good adherence I (discrimination and negative self-perception)

This session began with a discussion on their previous assignment, followed by how stigma and depression can act as barriers to good treatment adherence, their impacts, and the relevant rights and laws on HIV stigma and discrimination. Simple relaxation exercises were done, followed by role-play on the manifestation of stigma and depression, which enabled them to identify the negative cognition and self-perception in the roles. The adolescents played two short skits, each lasting less than five minutes; one was on stigma and the danger of discrimination, and the other was on identifying and dealing with negative self-perceptions regarding stigma, which was followed by a discussion on their reflections and lessons learned. They were also given brief group exercises to apply problem-solving techniques to resolve negative perceptions using a worksheet; the session ended with a recap of basic skills to address negative self-perception. Their take-home assignment was to identify other barriers to good medication adherence.

#### Session 3: Identifying and dealing with barriers to good adherence II (dealing with mood symptoms in relation to adherence)

This session began with a review of the previous week’s assignment, followed by training on mood-alleviating activities such as increasing pleasurable activities and mood monitoring. Participants were encouraged to participate in a dancing competition, introduced as a form of pleasurable activity during the session. After the session, they shared their emotions and feelings and were encouraged to identify and practice more pleasurable activities, such as dancing at home. In the second part of this session, they were also given thought record forms on which to capture, evaluate and restructure negative automatic thoughts, their homework being to keep a record of their thought processes.

#### Session 4: Handling privacy, social support, adjusting to HIV and antiretroviral treatment

This session dealt with issues relating to privacy, social support, adjusting to HIV status, and managing antiretroviral treatment in the context of their social life, family, and friends. The first part focused on reinforcing their ability to cope with their current way of life, stressors, and challenges. Participants were taught to identify, list, and annex the available social support around them. The second part started with giving information about medication side effects as barriers to good adherence. Next, they practiced adaptive coping techniques that enhance adherence, such as cue control and guided imagery, these strategies being role-played, the take-home assignment being to apply the problem-solving technique to address other barriers, such as forgetfulness. They also had group exercises that focused on identifying and rehearsing practical methods of maintaining good treatment adherence, such as short message services (SMS) with reminders, encouraging words, medication alarms, and how to use google calendar.

#### Session 5: Handling adherence slips and wrap up review

This session started with reviewing their homework. The final session focused on handling adherence slips (occasional medication skipping) and reviewed strategies to improve their quality of life, such as practicing healthy habits, e.g., exercise, music and dance, meditation, and positive thinking. The topics addressed during the previous four sessions were explicitly reviewed to bring the focus back to depression and adherence, with their data only being considered for analysis if they attended all the sessions.

##### Measures

Data were collected regarding their socio-demographic and clinical characteristics, with depression, adherence, and satisfaction levels being obtained by completing the Mini International Neuropsychiatric Interview for Children and Adolescents (MINI-KID), Patient Health Questionnaire (PHQ-9), Visual Analog Scale, and the Client Satisfaction Questionnaire, respectively.

###### Socio-demographic and clinical characteristics

The participants' socio-demographic characteristics included, age, gender, religion, ethnicity, education level, and parent's marital status. Data were also obtained on the type and level of social support, various clinical variables, e.g., mode of HIV transmission, age at first HIV diagnosis, and viral load, the clinical variables being retrieved from their medical records, with their and their parents' permission, where relevant.

###### Mini International Neuropsychiatric Interview for Children and Adolescents (MINI-KID)

The MINI-KID [24] is a brief structured clinical diagnostic interview designed to assess the existence of 24 ICD-10 and DSM-IV mental disorders comprehensively and concisely, and was used in conjunction with the PHQ-9 to screen the adolescent participants for depression.

###### Client Satisfaction Questionnaire (CSQ)

This self-administered tool assesses client satisfaction at the end the provision of services and enquires about respondents’ opinions and conclusions using options provided on a four-point scale. Examples include: “How satisfied are you with the amount of help you have received?”, the responses being 1 = " Quite dissatisfied", 2 = " Indifferent or mildly dissatisfied", 3 = " Mostly satisfied", 4 = " Very satisfied". To the question “Have the services you received helped you to deal more effectively with your problems?”, the options were, 4 = Yes, they helped a great deal”, 3 = ” Yes, they helped somewhat”, 2 = ” No, they didn’t help”, 1 = “No, they seemed to make things worse.” The scores are summed at the end of the questionnaire to form a single score measuring one dimension of overall satisfaction. The scores range from 8 to 32, with higher values indicating higher satisfaction and the tool has a good psychometric property, with the coefficient alpha ranging from 0.83 to 0.93.

###### The cognitive assessment tool-rapid version (CAT-rapid)

The CAT-rapid was developed by Joska et al. [25] and comprises four questions on cognitive symptoms, including registration of four words, a mini-trail-making test of four-letter/number pairs, and word recall. It is suitable for use among adolescents and young adults living with HIV [25]. It was used to screen for cognitive impairment in the participants.

##### Outcomes

For both groups, the outcomes of adherence and depression were measured at baseline and after five weeks of intervention, as well as at 24 weeks post-intervention in the intervention group, to assess the maintenance of the observed changes. Two tools were used: the visual Analog Scale (VAS) to determine treatment adherence and the Patient Health Questionnaire to measure depression severity.

Adherence was measured by the participants’ reports on the VAS, which is a direct consequence of attitudinal change toward medication use. The VAS is presented as a 10 cm long horizontal line [26], and calibrated by 5 cm, representing a scale from 0 to 100 (10 cm mark). Respondents have to indicate on the line where they think their adherence % rate is, with a score of less than 95% being defined as suboptimal [19]. Participants were also asked to indicate their level of adherence based on pill use in the past 30 days, which involved taking the correct pill dose at the appropriate time, as prescribed. This instrument has been widely used in both low- and middle-income countries and found to exhibit strong (r = 0.5–0.7) associations with other self-report measures, such as objective pill count, electronic drug monitor (EDM), and viral load (r = 0.35) [27].

Depression severity was measured with the Patient Health Questionnaire (PHQ-9), which is used to screen for depression and grade the severity of symptoms in general medical and mental health settings, having been validated for use in Botswana. It has nine items based on the Diagnostic and Statistical Manual of Mental Disorders, fourth edition (DSM-IV) criteria, and the scores range from “0” (not at all) to “3” (nearly every day), the cutoff of nine being consistent with a diagnosis of major depressive episodes in Botswana [28].

##### Data analysis

The socio-demographic and clinical characteristics were descriptively analysed to enable a comparison of the two groups, the outcomes being treatment adherence (measured using VAS) and depression (PHQ-9). Comparisons were made between the two groups based on an intention-to-treat sample, the data having been collected for baseline and at week five for both, and at 24 weeks follow-up for the intervention group. As the data were not normally distributed, the immediate post-intervention outcome was assessed with Rank Analysis of Covariance or Quade analysis of covariance (Quade ANCOVA) [29] of post-treatment scores, controlling for baseline scores. Partial eta squared (ƞ _p_^2^) effect sizes were calculated with 0.01, 0.06, and 0.14 representing small, medium, and large effect sizes, respectively [30]. Data for the intervention group at the 24-week follow-up were analyzed to assess the maintenance of the psychological therapy gains, this being done using changes in the depression and adherence scores over time within assigned groups using Friedman ANOVA. Post-hoc tests were performed with the Wilcoxon signed-rank test (using Bonferroni adjusted alpha value of 0.025) to determine maintenance of the treatment effect at 24 weeks. The effect size was calculated based on Wilcoxon signed-rank where $r=\left|\frac{z}{\sqrt{N}}\right|$, with the magnitude being based on Cohen’s classification of effect sizes, which is 0.1 for a small effect, 0.3 for a moderate effect, and 0.5 and above for a large effect.

## Results

### Socio-demographic and clinical characteristics

The study participants' age ranged from 15 to 19 years (Median = 18 years; IQR = 1.0), with 33 (66%) being females, the majority (86%) living with single parents and other relatives, most parents (81%) being currently separated or never married. Table 1 presents the socio-demographic and clinical characteristics at baseline, the two groups being similar. None of the participants reported any chronic neurological or medical condition, such as epilepsy or diabetes.

**Table 1:** Baseline Sociodemographic and clinical characteristics

Variables	Experimental Arm (n = 25)		Control Arm (n = 25)		Statistic	P-value
	n	%	n	%		
Gender					χ^2^	0.136
Male	11	44.0	6	24.0		
Female	14	56.0	19	76.0		
Age	Median = 18	IQR = 1	Median = 18	IQR = 2	*MWU*	0.579
Educational Attainment					χ^2^	0.089
Junior high School and less	9	36.0	15	60.0		
Senior high school and above	16	64.0	10	40.0		
Living arrangement					FET	0.221
Both parents	5	20.0	2	8.0		
Single parents and others	20	80.0	23	92.0		
Perceived support and counseling by the staff					χ^2^	0.152
Good	17	68.0	12	48.0		
Poor	8	32	13	52.0		
Cognitive screening score	Median = 10	IQR = 4	Median = 11	IQR = 3	*MWU*	0.143
Age of ART initiation	Median = 13	IQR = 15	Median = 15	IQR = 9	*MWU*	0.055
Mode of HIV infection					χ^2^	0.083
Perinatal infection	18	72.0	12	48.0		
Behavioral infection	7	28.0	13	52.0		
Family members living with HIV					FET	0.346
Yes	21	84.0	16	72.7		
No	4	16.0	6	27.3		

FET- Fisher’s Exact Test; MWU – Mann-Whitney U Test

### Effectiveness of the psychological intervention

The effectiveness of the intervention was measured by changes in the depression severity scores, as self-reported by the participants on the PHQ-9 tool, and treatment adherence by the VAS scores, as indicated in Table 2, from baseline to five weeks in the two groups. The Quade ANCOVA showed statistically significant differences in the post-intervention PHQ-9 and VAS scores when controlled for the pre-intervention or baseline scores. The intervention group scored significantly lower on depressive symptoms (PHQ-9 [F (1,50) = 12.0, p = 0.001, ƞ_p_^2^ = 0.20]) and higher on adherence scores (VAS [F (1,50) = 13.5, p = 0.001, ƞ_p_^2^ = 0.22]) than the control group, the effect sizes being large for both measures. The level of satisfaction with the intervention was observed to be high, with a median (IQR) score of 28 (2).

**Table 2:** Comparisons between intervention group and control on outcome measures

Variable	Intervention group (n = 25) Median (IQR)			Control group (n = 25) Median (IQR)			Statistics		
	Pre	Post	Difference	Pre	Post	Difference	F* value (1,50)	P value	Effect size (n_p_^2^)
PHQ-9	13(2.0)	7 (3.0)	6 (2.5)	13(3.0)	9 (2.0)	4 (2.5)	12.0	**0.001**	0.20
VAS	60(30.0)	80 (30.0)	20 (5.0)	70 (20.0)	80(18)	10 (10.0)	13.5	**0.001**	0.22

F* Quade’s ANCOVA, Significant p values in bold

### Maintenance of psychological therapy gains

The percentage of change in median scores from baseline to 24-week follow-up for the treatment group is shown in Table 3, with the Friedman ANOVA test indicating that there was a significant difference in PHQ-9 score across the three-time points (baseline, 5-weeks, and 24 weeks follow-up [χ^2^ (2, n = 21) = 37.95, p < 0.05]). Inspection of the median values for the PHQ-9 score showed a decrease in depression score from baseline (Median [*Md*] = 13.0) to five weeks (*Md* = 7) and a further decrease at 24 weeks follow-up (*Md* = 6.0). In addition, for adherence, the Friedman ANOVA showed a significant change in VAS score across the three-time points [χ^2^ (2, n = 21) = 36.69, p < 0.05], while the median value for adherence increased from the baseline (Md = 60.0) to post-intervention (Md = 90.0) but was the same at follow-up (Md = 90.0).

**Table 3:** End points comparison between time points within experimental group

Measure	Baseline (n = 25)	Post intervention (n = 25)				24 weeks follow up (n = 21)				Effect size r
	Median (IQR)	Median (IQR)	% Change	z	p	Median (IQR)	% Change	z	p	
PHQ-9	13 (2.0)	7 (2.5)	46.2	− 4.27	** < 0.01**	6 (1.5)	53.8	− 4.03	** < 0.01**	0.88
VAS	60 (20)	80 (30.0)	33.3	− 4.40	** < 0.01**	90 (12.5)	50	− 4.05	** < 0.01**	0.88

Significant p values in bold

The post-hoc tests (Wilcoxon signed-rank test) showed that the significant improvements in depressive symptoms (*z* =  − 4.03, *p* =  < 0.01, *r* [effect size] = 0.88) and adherence (*z* =  − 4.05, *p* =  < 0.01, *r* = 0.88), and the large effect sizes found at post-test in the intervention group were well maintained at 24 weeks.

## Discussion

This study aimed to explore the effectiveness of PI on depression and treatment adherence in a sample of ALWHIV in Botswana and, to the best of our knowledge, is the first of such studies in this sub-population. The study's findings demonstrated that a short course of problem-solving and psychoeducation was effective in reducing depressive symptoms and improving adherence in ALWHIV. After a 5-week program, the intervention group with mild to moderate depression showed a reduction in symptoms compared to the control group while controlling for baseline scores, the improvements being maintained at a 24-week follow-up for depression and adherence, with participants being highly satisfied.

Our study contributes to the existing literature on the well-being of ALWHIV in several ways. Firstly, it illustrates the cross-cultural relevance of a western-based intervention, adapted to local contexts, to a population that is different from the original one for which it was developed, thereby serving as a form of external validation of the psychological intervention. Secondly, it provides preliminary data on the effectiveness of a brief intervention, which has significant implications for cost-effectiveness, man-hours expended, and interruption to patients’ lives. Finally, this is the first study to explore the effectiveness of a multicomponent intervention in the ALWHIV in Botswana with a high burden of HIV. It reinforces the existing view that multicomponent psychological interventions for ALWHIV may be preferable to single-component forms, such as text message reminders, which are also effective [7, 8].

Our findings compare reasonably with the studies conducted in other parts of the world [7, 8]. As observed, the median differences in both depressive symptoms and treatment adherence scores were significantly larger for the intervention group than the controls, with the former group showing improvements in both. In line with Mimiaga et al. [8], the present study suggests that problem-solving and psychoeducation are effective coping tools for ALWHIV in Botswana. Safren and colleagues [7] reported the effectiveness of problem-solving in the reduction of depressive symptoms and improved adherence among adolescents and young adults, in agreement with the current study.

The intervention group maintained the treatment effect with significantly higher adherence and lower depression scores 24 weeks post-intervention than the baseline scores. Although the follow-up duration was relatively short, this finding further supports the need for integrating psychological interventions in promoting adherence to treatment among individuals living with HIV [7]. However, since the present study could not conclude on the long-term effect of this intervention as was done by a previous study that followed up the participants for one year [7], further longitudinal research with more robust sample sizes is needed.

Unlike the study by Safren and colleagues [7], which exposed the participants to eleven weeks of intervention, the present research used five weeks among ALWHIV. While Mimiaga et al. [8] did not measure or relate depression with adherence, they also reported a significant improvement in adherence scores among ALWHIV using five-week multicomponent psychological strategies, including problem-solving rehearsal and psychoeducation. In support of Mimiaga et al. [8], the present study suggests that a brief course of therapy can also be effective, as has been found in longer durations of therapy sessions in adolescents. In addition, the shorter sessions could conceivably fit better into adolescents’ school schedules with minimum academic work disruption.

From a theoretical perspective, the positive effect of a depression-targeted intervention in improving medication adherence in ALWHIV could be premised on the contention that depression significantly affects medication adherence, with Davidson noting that poor adherence is related to the ‘weakness of will’ [31, 32]. Invariably, an illness that weakens willpower and impairs judgment, such as depression, can affect adherence, hence the focus on depression in this manuscript. Moreover, the concept of foresight, hypothesized by Reach [33], gives priority to motivations that are focused on the direction of the future. Depression is characterized by hopelessness and a negative view of the future [34], and in the absence of motivations that are focused on the future, patients would naturally be non-adherent to their prescribed treatment [33]. Therefore, restoration of hope and the inclusion of realistic and potentially achievable sub-goals in therapy can potentially increase adherence in ALWHIV [35].

This study adapted an existing psychological intervention program designed in high-income countries, such as the United States of America, and used role play, locally relevant, and adolescent-friendly video vignettes for demonstration and psychoeducation. These were done in the local language, Setswana, mixed with English, with locally applicable and feasible illustrations and discussions being used in the problem-solving session. The other components included SMS with reminders, encouraging words, and using google calendar/alarms. While the present study did not compare the effect of a single component with the multicomponent method, it reinforces the claim by Safren and colleagues that the latter is more appropriate in some contexts [7]. In addition, it addresses both the psychosocial needs and other barriers related to poor adherence, such as forgetfulness and issues regarding medication side effects. Finally, as with previous studies [7, 8], the adolescent participants understood the sessions well, this claim being based on the subjective observation of good participation in discussions before and after every session, compliance with the home or in-session exercises, and the high median satisfaction score from the feedback forms.

### Limitations

A number of limitations may have affected the present study, the first being the small sample size, suggesting the need for further studies with larger samples of ALWHIV to establish the efficacy of this treatment modality. Second, the effectiveness of this intervention is limited to mild-to-moderate depression, as participants with severe forms were excluded from this study. Therefore, it is unclear if and how the findings may be generalized to those patients, with extensions to that group being made with caution. Third, the reliability of the VAS in assessing adherence may be low and subjective compared to other methods, such as electronic pill caps (MEMS, AARDEX) [26], which suggests a further cautious interpretation of the study's findings. However, the VAS was moderately correlated with the viral load (ρ = − 0.31; p < 0.01), as indicated in the first phase of the study conducted among 622 ALWHIV. The self-report nature of the study may also have introduced a recall bias and inaccurate reporting of symptoms.

It should also be noted that the amount of time spent, frequency of meetings, and other non-specific effects of engaging with the intervention group may have contributed to some of the improvements noted, highlighting the need for better controls in future studies, such as befriending and attending to both groups weekly. The authors could not comment on the acceptability and suitability of the intervention based on the mode of infection (MOI), although the two groups were similar in this regard, its influence was controlled for. There may be other factors peculiar to this setting that were not controlled for, hence the need for a cautious interpretation of this report. Finally, the study could not conclude on this intervention's long-term effect as the follow-up duration was relatively short; further studies with longer follow-up periods are warranted in this regard.

## Conclusions

This study has shown that a relatively short multicomponent psychological intervention that consists of problem-solving, psychoeducation, and rehearsal is effective in reducing depressive symptoms and improving adherence among adolescents infected with HIV in the short term. This has significant implications for the cost-effectiveness of treatment approaches as well as care policies and planning in resource-constrained settings, such as in Botswana and many African countries. With Africa being the global epicenter of the HIV pandemic, and the high prevalence rates, specifically among young adults, being well reported to affect depression and treatment uptake, finding low-cost interventions that can improve the quality of life of those infected remains a priority for all areas of public health research. Studies are suggested with longer follow-ups to provide a firmer basis for these interventions' long-term benefits. As the mental health and well-being of ALWHIV continue to attract public mental health attention, studies providing evidence for effective adjunct treatments remain critical to improving treatment outcomes among this young at-risk cohort.

## Data Availability

The datasets used and analyzed during the current study are available from the corresponding author upon reasonable request.

## References

[1] Zhou S, Cluver L, Shenderovich Y, Toska E (2021). Uncovering ART adherence inconsistencies: an assessment of sustained adherence among adolescents in South Africa. J Int AIDS Soc.

[2] Ekat MH, Yotebieng M, Leroy V, Mpody C, Diafouka M, Loubaki G, Mahambou-Nsondé D, Ibara BRO, Bernard C, Sabin C (2020). Association between depressive symptoms and adherence among adolescents living with HIV in the Republic of Congo: a cross sectional study. Medicine.

[3] Sekhon S, Marwaha R. Depressive cognitive disorders. 2020. 32644682

[4] Sahin S, Önal TO, Cinar N, Bozdemir M, Cubuk R, Karsidag S (2017). Distinguishing depressive pseudodementia from Alzheimer disease: a comparative study of hippocampal volumetry and cognitive tests. Dementia Geriatric Cognitive Disorders Extra.

[5] Haji H (2018). Factors associated with poor adherence to ant-retroviral therapy among HIV/AIDS patients attending care and treatment clinic at Mnazi Mmoja Hospital. Zanzibar Int J Infect Dis.

[6] Murray KR, Dulli LS, Ridgeway K, Dal Santo L (2017). Improving retention in HIV care among adolescents and adults in low-and middle-income countries: a systematic review of the literature. PLoS ONE.

[7] Safren SA, Bedoya CA, O’Cleirigh C, Biello KB, Pinkston MM, Stein MD, Traeger L, Kojic E, Robbins GK, Lerner JA (2016). Treating depression and adherence (CBT-AD) in patients with HIV in care: A three-arm randomized controlled trial. Lancet HIV.

[8] Mimiaga MJ, Bogart LM, Thurston IB, Santostefano CM, Closson EF, Skeer MR, Biello KB, Safren SA (2019). Positive strategies to enhance problem-solving skills (STEPS): a pilot randomized, controlled trial of a multicomponent, technology-enhanced, customizable antiretroviral adherence intervention for HIV-infected adolescents and young adults. AIDS Patient Care STDS.

[9] Olashore AA, Paruk S, Akanni OO, Tomita A, Chiliza B (2021). Psychiatric disorders in adolescents living with HIV and association with antiretroviral therapy adherence in sub-Saharan Africa: a systematic review and meta-analysis. AIDS Behav.

[10] Garvie PA, Lensing S, Rai SN (2007). Efficacy of a pill-swallowing training intervention to improve antiretroviral medication adherence in pediatric patients with HIV/AIDS. Pediatrics.

[11] Dowshen N, Kuhns LM, Johnson A, Holoyda BJ, Garofalo R (2012). Improving adherence to antiretroviral therapy for youth living with HIV/AIDS: a pilot study using personalized, interactive, daily text message reminders. J Med Internet Res.

[12] Shegog R, Markham CM, Leonard AD, Bui TC, Paul ME (2012). “+ CLICK”: pilot of a web-based training program to enhance ART adherence among HIV-positive youth. AIDS Care.

[13] Lyon ME, Trexler C, Akpan-Townsend C, Pao M, Selden K, Fletcher J, Addlestone IC, D'Angelo LJ (2003). A family group approach to increasing adherence to therapy in HIV-infected youths: results of a pilot project. AIDS Patient Care STDS.

[14] Gonzalez JS, Batchelder AW, Psaros C, Safren SA (2011). Depression and HIV/AIDS treatment nonadherence: a review and meta-analysis. J Acquired Immune Deficien Syndr.

[15] Blashill AJ, Perry N, Safren SA (2011). Mental health: A focus on stress, coping, and mental illness as it relates to treatment retention, adherence, and other health outcomes. Curr HIV/AIDS Rep.

[16] Ironson G, O’cleirigh C, Kumar M, Kaplan L, Balbin E, Kelsch C, Fletcher MA, Schneiderman N (2015). Psychosocial and neurohormonal predictors of HIV disease progression (CD4 cells and viral load): a 4 year prospective study. AIDS Behav.

[17] Olashore AA, Paruk S, Tshume O, Chiliza B (2022). Depression and suicidal behavior among adolescents living with HIV in Botswana: a cross-sectional study. Child Adolesc Psychiatry Ment Health.

[18] Dick B, Ferguson BJ (2015). Health for the world's adolescents: a second chance in the second decade. J Adolesc Health.

[19] Mai HT, Le GM, Tran BX, Do HN, Latkin CA, Nguyen LT, Thai TPT, Le HT, Ngo AT, Nguyen CT (2018). Adherence to antiretroviral therapy among HIV/AIDS patients in the context of early treatment initiation in Vietnam. Patient Prefer Adherence.

[20] Whitley E, Ball J (2002). Statistics review 4: sample size calculations. Crit Care.

[21] Kadam P, Bhalerao S (2010). Sample size calculation. Int J Ayurveda Res.

[22] CfIOoM S (2002). International ethical guidelines for biomedical research involving human subjects. Bull Med Ethics.

[23] Gunthorp J. Age of consent: legal review In: Botswana country report. Botswana Thomson Reuters Foundation

[24] Sheehan DV, Sheehan KH, Shytle RD, Janavs J, Bannon Y, Rogers JE, Milo KM, Stock SL, Wilkinson B (2010). Reliability and validity of the mini international neuropsychiatric interview for children and adolescents (MINI-KID). J Clin Psychiatry.

[25] Joska JA, Westgarth-Taylor J, Hoare J, Thomas KG, Paul R, Myer L, Stein DJ (2011). Validity of the international HIV dementia scale in South Africa. AIDS Patient Care STDS.

[26] Wewers ME, Lowe NK (1990). A critical review of visual analogue scales in the measurement of clinical phenomena. Res Nurs Health.

[27] Finitsis DJ, Pellowski JA, Huedo-Medina TB, Fox MC, Kalichman SC (2016). Visual analogue scale (VAS) measurement of antiretroviral adherence in people living with HIV (PLWH): a meta-analysis. J Behav Med.

[28] Molebatsi K, Motlhatlhedi K, Wambua GN (2020). The validity and reliability of the Patient Health Questionnaire-9 for screening depression in primary health care patients in Botswana. BMC Psychiatry.

[29] Quade D (1967). Rank analysis of covariance. J Am Stat Assoc.

[30] Cohen J (1992). Statistical power analysis. Curr Dir Psychol Sci.

[31] Davidson D, Feinberg J (1970). How is weakness of will possible?’. Moral Concepts.

[32] Davidson D, Wollheim R, Hopkins J (1982). Paradoxes of irrationality. Philosophical Essays on.

[33] Reach G (2008). A novel conceptual framework for understanding the mechanism of adherence to long term therapies. Patient Prefer Adherence.

[34] Beck AT (1967). Depression: Clinical, experimental, and theoretical aspects.

[35] Meichenbaum D, Turk DC (1987). Facilitating treatment adherence: A practitioner's guidebook.

